# Our Health, Our Action, Our Planet—a Call to Action for Microbiologists To Engage in Climate Research

**DOI:** 10.1128/mBio.02502-21

**Published:** 2021-09-07

**Authors:** Nguyen K. Nguyen, Arturo Casadevall

**Affiliations:** a American Society for Microbiology, Washington, DC, USA; b Johns Hopkins Bloomberg School of Public Health, Baltimore, Maryland, USA

## EDITORIAL

Imagine a world where both the geographical distribution and incidence of plant, animal, and human pathogens have increased by several magnitudes compared to the COVID-19 pandemic because we failed to limit global temperature rise below 1.5°C of the preindustrial levels. In that world, we, humans, would suffer tremendously, and the altered environment could pose an existential threat to our species. Today, we are on the edge of that precipice and facing the last chance to prevent irreversible climate damages from occurring. Even though the world is still fighting the COVID-19 pandemic, the threats due to climate change require urgent and definitive actions from *us* now before it is too late.

Historically, despite the efforts of many to highlight the problem, climate change was not a top issue for health professionals, including us, microbiologists. As world leaders plan a series of meetings this fall to develop collective actions to address climate change, ASM Journals is joining other leading health journals to publish the editorial in this issue that calls for emergency actions to limit global temperature increases, restore biodiversity, and protect health (1). Not only does our support for this joint editorial aim to raise awareness of the increasingly dire situation, but it is also our call for the microbial sciences community to commit and contribute to advance our understanding of climate change, microbial systems, and their relationship to various aspects of human well-being.

As microbiologists, we can and should play leading roles in contributing to the global efforts to address climate change. Microbes act as modulators of carbon in the biosphere. The effects of microbes on climate change and climate change on microbes are well recognized but often overlooked. From basic microbial processes such as influencing carbon release from arctic tundra to capturing carbon as CO_2_ and CH_4_, the impacts of microbial systems on climate change and subsequently to human health are enormous and complex and require further research. Furthermore, the effects of human activities such as population growth, urbanizations, migrations, etc., on microorganisms and anthropogenic climate change require large-scale studies involving multiple fields, with the goal of collaborating to create sustainable and practical solutions to address this potential existential crisis.

We must do more. The release of the joint editorial is a start (1), but this is a minuscule step considering the task ahead. We are excited to see the commitment from ASM to address climate change. Two months ago, the American Academy of Microbiology (Academy), the honorific leadership group and scientific think tank at ASM, set a goal that for the next 5 years it would focus on promoting the understanding of the relationship between microbes and climate change and building a scientific framework to inform climate change policies and market innovations. This audacious goal was set in response to the feedback of the Academy fellows who overwhelmingly supported climate change as the topic of focus. We realize microbiologists can’t do this alone, but microbiologists can make a critical contribution to understanding the effects on earth life and perhaps help in providing new solutions. We look forward to partnering with our colleagues of diverse expertise and sectors in this journey to solve one of the biggest challenges for humanity.

President Abraham Lincoln once said, “The best way to predict your future is to create it.” For climate change, we used to ask, “What future do we want for our children?” However, that future is nearer than we may have thought of. Without substantial investments and efforts to reach and sustain net zero of global anthropogenic CO_2_ emission, the global temperature could reach the 1.5°C mark as early as 2030. Therefore, the question now is, “What future are we willing to create for ourselves?” We must do more.
