# Erratum for Nguyen and Casadevall, “Our Health, Our Action, Our Planet—a Call to Action for Microbiologists To Engage in Climate Research”

**DOI:** 10.1128/mbio.00015-22

**Published:** 2022-02-08

**Authors:** Nguyen K. Nguyen, Arturo Casadevall

**Affiliations:** a American Society for Microbiology, Washington, DC, USA; b Johns Hopkins Bloomberg School of Public Health, Baltimore, Maryland, USA

## ERRATUM

Volume 12, no. 5, e02502-21, 2021, https://doi.org/10.1128/mBio.02502-21. The author byline should appear as shown in this erratum.

